# Potential effects of cinnamon on cancer prevention and progression

**DOI:** 10.3389/fnut.2025.1717834

**Published:** 2025-12-11

**Authors:** Madison Anderson, William Hrivnak, Khanneth Prak, Amy Stockert

**Affiliations:** Department of Pharmaceutical and Biomedical Sciences, Ohio Northern University, Ada, OH, United States

**Keywords:** nuclear factor kappa B, NFκB, HIF1-VEGF pathway, Nrf2, MAPKs, cinnamaldehyde, cinnamic acid, cinnamyl acetate

## Abstract

Cinnamon has been used medicinally for centuries, but recently *in vitro* research has suggested it may have a role in cancer prevention and potentially treatment. The search for alternative and subjunctive therapies is essential due to the public demand and the increasing cost of healthcare. Here we review the biologically active components of cinnamon and discuss the methods of potential cinnamon activity against cancer, including: transcription factor regulation and kinase activity. Nuclear Factor kappa B (NFκB) is a stress sensitive transcription factor that regulates transcription of genes involved in tumor progression and is inhibited by cinnamon components. Another way that cinnamon inhibits tumor growth is by suppression of transcription factor activator protein 1 (AP1) which interacts with genes responsible for apoptosis, metastasis and inflammation. Hypoxia-inducible transcription factor 1 (HIF1) and vascular endothelial growth factor (VEGF) are involved in angiogenesis, especially in the tumor microenvironment. The HIF1-VEGF pathway is a target of cinnamaldehyde, a compound found in cinnamon. Nuclear factor erythroid related factor 2 (Nrf2) is also examined and has been indicated to affect cancer progression and potentially provide preventative measures; various cinnamon derivatives target Nrf2. A cinnamaldehyde derivative has been implicated in a reduction of the mitogen-activated protein kinases (MAPKs), which are a group of kinases that regulate proliferation. Additionally, cinnamon components have been tied to cancer prevention by positively affecting the gut microbiome and inhibiting inflammation. The review concludes with a discussion of the future research needed, including the need for clinical studies and potential risk associated with cinnamon intake.

## 1 Introduction

Cinnamon is one of the oldest known spices and has been used medicinally for gastrointestinal discomfort, as well as other ailments for centuries (1–3). Within the last few decades, cinnamon has been explored, albeit with controversy, as an alternative or subjunctive therapy for patients with type 2 diabetes mellitus. Numerous studies highlight its anti-diabetic properties but have yet to demonstrate with certainty a mechanism of action (4–6). Controversy stems from the few studies that do not support its antidiabetic effects, which in some cases can be partially explained by population differences in the study group (7). Cinnamon has also been shown with little controversy to improve the lipid profiles in patients, including those with diabetes and heart disease (8).

Recently cinnamon has been studied for its anti-cancer effects that likely stem both from its chemical composition as well as some of the polyphenols commonly found in a variety of cinnamon species (9). As healthcare costs continue to expand and the popularity of alternative medicine gains attention, it is important to include evaluations of the effects of these plant-based medicines that have been shown to be effective against a variety of chronic diseases including cancer, diabetes, and cardiovascular disease (8, 10–12). An excess of half of the drugs used in the past two decades are either directly derived from plants or altered chemically from plants (3). Many patients want options and alternatives in cancer therapies, therefore research that considers potential mechanisms are an important part of determining which alternative should be selected for clinical research. Clinical research is not only important for evaluating efficacy but also considering safety. This review will first discuss the biologically active cinnamon compounds then discuss a selection of recent cinnamon and cancer studies organized by potential mechanism of action including the targeting various transcription factors and kinases. It is important to realize that although these are organized based on the potential target of the cinnamon compound, many times, due to the complexity of the cancer disease state, multiple targets will be overlapping.

## 2 Biologically active cinnamon compounds

Cinnamon contains several compounds that are biologically active, although some have been studied better than others. Cinnamaldehyde has been examined extensively and has been reported to target several nuclear receptors as well as transcription factors within a variety of signaling pathways (13). Cinnamic acid, cinnamyl acetate, coumarin, caffeic acid are also important components (10, 14). In addition to these compounds cinnamon also contains several polyphenols that have suspected biological activity. These include catechin-based structures and procyanidin B and A-type linkages of polyphenolic compounds, resulting in a large variety of polyphenol-based components (11, 15). Polyphenolic compounds such as these found in cinnamon have been studied for their anticancer potential in a number of pathways and transcription factors including the NFκB and Nrf2 transcription factors and signaling pathway MAPK (16).

Within many studies, cinnamaldehyde has been used to contribute to apoptosis in cancer cells by acting on different mechanisms (13, 17). A study conducted by Liu et al. screens targets for cinnamon in the treatment of breast cancer and explores therapeutic mechanisms (13). The active ingredients of cinnamon were screened based on their oral bioavailability (OB) and through the Lipinski rule-based drug-likeness (DL) (13). A total of 147 active compounds were collected but 12 were selected based on their DL and OB. Out of the 12, oleic acid, di-isobutyl phthalate (DIBP), and cinnamaldehyde were identified to be the most critical ingredients. These compounds are used to target different nuclear receptors of cancer which includes peroxisome proliferator-activated receptor gamma (PPARγ), toll-like receptor 4 (TLR4), brain-derived neurotrophic factor (BDNF), and peroxisome proliferator-activated receptor alpha (PPARα) (13). These are involved affecting lipid metabolism, glucose homeostasis, and tumor progression within breast cancer (13).

In another study conducted by Gopalakrishnan et al., the aim was to demonstrate proteasome-inhibiting and pro-apoptotic actions of procyanidin-B2 (PCB2), which is a component of cinnamon-extracted proanthocyanins (18). The study extracted PCB2 by grinding, vortexing, and filtering cinnamon into an aqueous extract called aqueous cinnamon extract (ACNE). The sample was then measured by 20S proteasome assays, isolation and assay for endogenous 26S proteasome activity, and other different methods to measure and observe suppressive catalytic activity of proteasome. PCB2 has shown to have anti-proliferative and pro-apoptotic activity *in vitro* within cancer cells and inhibits catalytic activity of proteasomes decreasing anti-apoptotic markers allowing apoptosis within cancer cells (18).

Cinnamon is a natural aromatic carboxylic acid containing an acrylic acid group allowing cis or trans configuration (18, 19). Adding functional groups to different positions of the aromatic carboxylic acid allows different actions for dealing with cancer. A review from Ruwizhi and Aderibige compiled studies that focus on structural differences from cinnamon derivatives and their effects on different disease states. For cancer, there are many different *in vitro* studies that represent different active compounds and their effects on different cancer variations. One study within the review synthesized cinnamic acid derivatives with 1,2,3-triazolic moiety and observed their antimetastatic activity (19, 20). This resulted in proliferation impairment and decreases in invasion and adhesion of cancer cells against colon carcinoma and lung adenocarcinoma cells (19, 20). Another study within the review synthesized novel oleanolic acid (OA)-cinnamic acid ester and glycyrrhetinic acid (GA)-cinnamic acid ester derivatives and access their cytotoxicity against MCF7(breast cancer), HeLa (cervical cancer), and LO2 (normal hepatic cells). The result includes inducing apoptosis and increasing reactive oxygen species in HeLa and improving inhibition of MCF7 (19, 21).

An important active compound within cinnamon is eugenol which induces apoptosis in promyelocytic leukemia cells (HL60) (22). It also increases reactive oxygen species (ROS) within colon cancer, allowing it to be an alternative treatment option (22). Cinnamaldehyde also shows evidence of inducing apoptosis for HL60 but, at high concentrations, it upregulates CD95 expression and poly (ADP-ribose) polymerase (PARP) cleavage (22). At low concentrations, cinnamaldehyde induces apoptosis by upregulating caspase-8 activity, Bax, and Bid decreasing antiapoptotic proteins (22).

## 3 Potential mechanisms of action

### 3.1 NFκB is inhibited by cinnamon components

**Figure 1:**
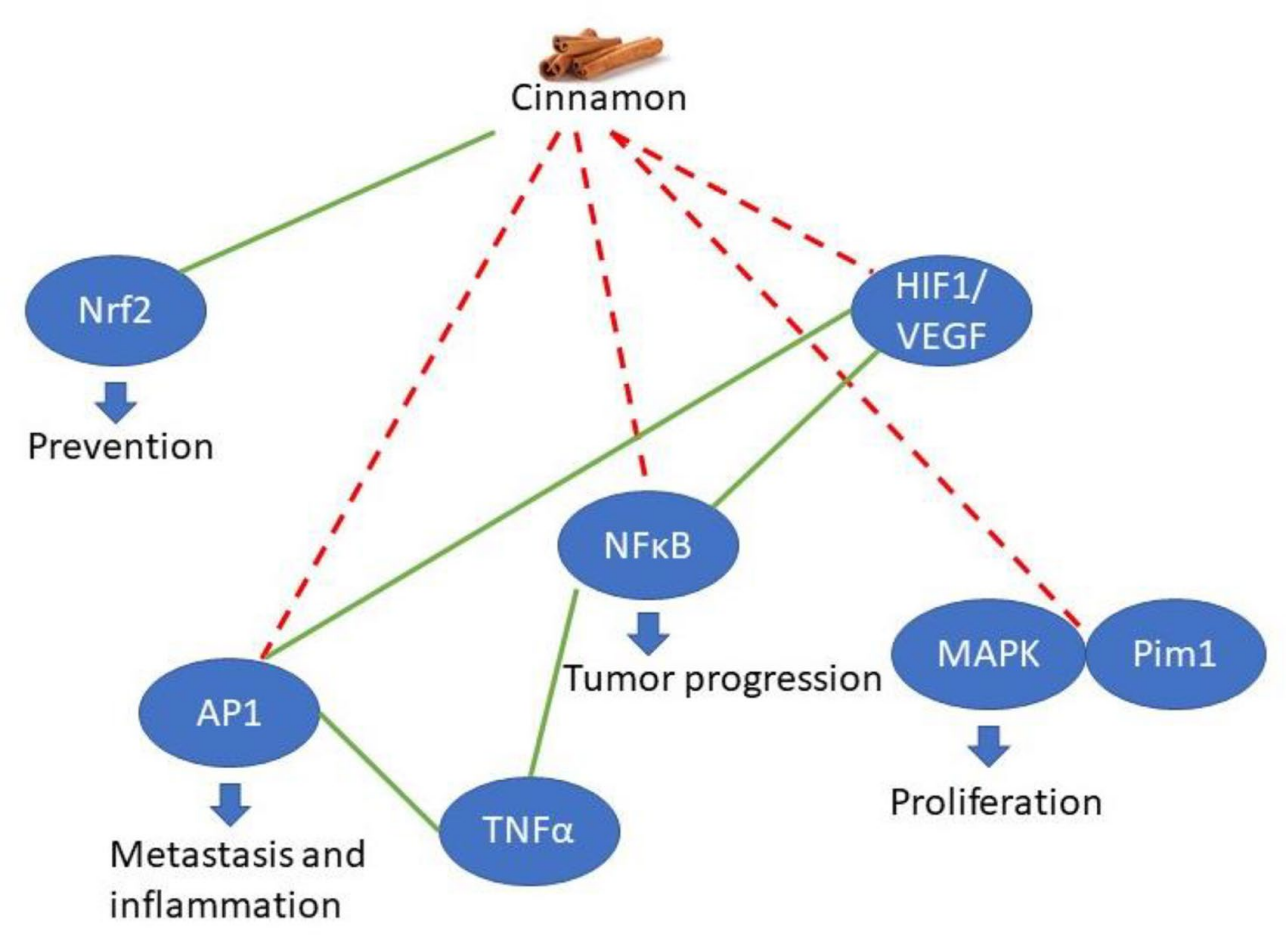
Key relationships of cinnamon components on cancer. Green lines indicate an activation and dashed red lines indicate inhibition. Overall cinnamon enhances prevention and inhibits targets that promote cancer.

Nuclear Factor Kappa B (NFκB) functions as a transcription factor that is stress sensitive. Endogenous levels of NFκB regulate transcription in response to the stress stimulus including those from tumor necrosis factor alpha (TNFα), reactive oxygen species, inflammatory factors and infection. Increases in stress stimuli or NFκB levels are often observed in cancer. NFκB is involved in tumor progression, metastasis, and drug resistance (23–26). Generally speaking, the involvement of cinnamon with NFκB is inhibitory in nature which has been demonstrated in human studies (27). NFκB can be excluded from the nucleus when it interacts with inhibitory proteins known as IκBs, which interact with NFκB and mark it for degradation (28). IκB proteins are phosphorylated by IκB kinase (IKK), leading to their degradation and subsequent nuclear translocation of NFκB. Certain polyphenols, such as quercetin and catechin derivatives, inhibit IKK, thereby blocking IκB phosphorylation and NFκB activation (29). Polyphenol modulation of NFκB comes from both the inhibition of IKK activation which prevents IκB phosphorylation and subsequent degradation. This results in inhibited NFκB DNA binding activity making cinnamon a potential anticancer treatment that should be further explored in clinical trials (16). Key relationships are summarized in Figure 1.

There are at least two possible pathways of NFκB that are involved in cancer. One is within the canonical NFκB pathway. There is an increase of mutation rates of reactive oxygen species (ROS) and reactive nitrogen intermediates (RNI) promoting tumor initiation (30, 31). Promotion of angiogenesis allows tumor invasion by regulating pro-angiogenic genes like vascular endothelial growth factor (VEGF) or macrophage inflammatory protein 1 (MCP1) (30). Proinflammatory cytokines such as TNFα and IL1β activates the canonical pathway resulting in expression of anti-apoptotic genes promoting tumor survival and progression of cancer (30). It is also involved in the inflammatory mechanism and modulates innate immunity (31). A second possible cancer pathway is the non-canonical NFκB pathway. By modulating anti-cancer immune response by recruiting and regulating immune cells, the NFκB pathway plays another role in cancer development (30). This is done by promoting tertiary lymphoid organs (TLOs) formation through lymphotoxin beta receptor (LTβR) inducing expression of adhesion molecules and chemokines (30). The non-canonical NFκB pathway is also involved in lymphoid organ development and adaptive immunity.

A review conducted by Wu et al., recorded and observed microRNA (miRNA) activity with NFκB within cancer cells. MicroRNA is an endogenous single-stranded RNA which affect both NFκB signaling and immune responses. These miRNA-NFκB effects play into tumorigenesis and progression by regulating expression of oncogenes or tumor suppressors (23). The polyphenols such as those found in cinnamon have been found to regulate a variety of miRNAs involved in insulin resistance and obesity (32, 33). Additional studies looking at the effects of cinnamon on cancer related miRNAs are essential to the future of the field of complementary and alternative medicine (CAM). Within ovarian cancer proliferation, low expression of miR9 promotes NFκB1 expression enhancing NFκB activity (34, 35). NFκB activation can result in resistance to cancer therapy. It does so by activating TNF-related apoptosis-inducing ligand (TRAIL), inducing NFκB-dependent overexpression of miR21 and miR100 and targeting TNF receptor-associated factor 6 (TRAF6). This effect leads to increased activation of NFκB (23, 36–38). Radioresistance occurs by NFκB activation when miR668 targets IκBα (an inhibitor of NFκB) activating NFκB shown in MCF7 and T47D cells (39). There are different treatment strategies that target miRNA such as antagonists and modified chemically antisense oligonucleotides decreasing expression of miRNA (23). This summarizes just a selection of miRNA effects on NFκB observed in *in vitro* studies.

NFκB inhibitors can be effective for the treatment of lung cancer. As discussed previously, NFκB upregulates genes involved in cell proliferation, metastasis, angiogenesis, and suppression of apoptosis (40). Other mechanisms also exist. This includes expression of bromodomain-containing protein 4 (Brd4), which maintains active lung cancer cells. Superoxide dismutase 2 (SOD2) can also induce action and increase IκB Kinase beta (IKKβ) transcription. In addition, tumor necrosis factor receptor associated factor 6 (TRAF6) contributes to pathogenesis of lung cancer (41, 42). The study predicts that utilizing TRAF6 can reduce activation and suppress lung tumorigenesis (31).

One study conducted by Agrawal et al., evaluated the expression of in breast cancer and its association with estrogen receptors (ER) and progesterone receptors (PR). The study resulted in an altered expression of expression in solid tumors and hematopoietic proliferations (43, 44). Cyclin D1 was induced by allowing development of normal mammary glands and breast cancer (45). This occurs due to cyclin-dependent kinases (cdks) binding with ckd6 and ckd4 causing phosphorylation to retinoblastoma (RB) proteins (31). This prevents suppression of E2F transcription leading to transcription of other genes causing cellular proliferation (31). A total of 119 patients with breast cancer had cells collected by Patey’s conservative radical mastectomy and then underwent immunohistochemical staining. As a result, expression was higher in breast cancer when compared to controlled normal breast cells (45).

### 3.2 Activator protein 1 activity is blunted by cinnamon components

The AP1 transcription factor has become an area of interest in cancer research due to its involvement in these tumorigenic pathways. Pharmacologic agents that target AP1 could be useful in treating cancer by inducing apoptosis, inhibiting metastasis, and reducing inflammation. Research has found decreased activity of AP1 through the use of phytochemicals from various herbal sources such as retinoids, flavonoids, viscolin, curcumin, and cinnamon. These compounds work through several different mechanisms such as suppression of signaling pathways, reduction in AP1 levels, and prevention of DNA binding. Some compounds also inhibited NFκB activity in addition to AP1 (46). AP1 controls several genes related to survival, proliferation, and differentiation of cells (47, 48).

In cancerous cells, dysregulation of oncogenes and the release of pro-inflammatory cytokines such as TNFα increase AP1 activity, which facilitates the proliferation of tumors and the metastasis of cancer cells to other tissues (49). AP1 is also closely tied to several other species involved in tumorigenesis. AP1 is almost always activated by the same stimuli as NFκB which has similar roles involved with regulating growth, apoptosis, and inflammation (50). Additionally, AP1 regulates genes that are responsible for TNFα and interleukin-1, which can further promote inflammation. Lastly, AP1 can contribute to angiogenesis of tumors through its association with HIF1α (49). The involvement of AP1 in cancer is therefore overlapping with other mechanisms. Few studies have been completed looking at the effects of cinnamon with AP1, but those few show promise and highlight a need for additional research including more clinical studies.

A study conducted in *in vivo* mouse models investigated the effects of cinnamon on NFκB and AP1. Six-week-old male mice were inoculated with a mouse melanoma cell line and divided into a treatment group and a control group. The treatment group received 10 mg of cinnamon extract by mouth per dose while the control group received a placebo by mouth. Mice were treated for 30 days after which tumor size was measured, protein expression was analyzed by Western blot, and RNA expression was analyzed by RT-PCR. Mice in the treatment group had smaller tumors by mass (*p* < 0.005), decreased expression of NFκB and AP1 (*p* < 0.005 for both), and decreased expression of their target genes Bcl-2 and BcL-XL (*p* < 0.005). The results of this study show that AP1 may be an important pathway utilized by the compounds in cinnamon for inducing apoptosis in cancer cells and that further research investigating AP1 may be beneficial for understanding cinnamon’s role in cancer treatment (51, 52).

Another study concluded that trans-cinnamaldehyde and p-cymene provide a strong anti-inflammatory that can function by inhibiting transcription of NFκB and AP1 by inhibiting the mitogen-activated protein kinase (MAPK) pathway. Reduction in NFΚB and AP1 reduce the expression of several pro-inflammatory cytokines including TNFα (53).

### 3.3 Nrf2 activation may inhibit cancer development but enhance growth of existing tumors

Nuclear factor erythroid-related factor 2 (Nrf2) is a regulator of transcription that controls the expression of a variety of genes which when expressed protect the cell from toxins and oxidative stress. Activation of Nrf2 may reduce cancer risk and is discussed further in the cancer prevention section of this review. It has also been shown that tumors exhibit elevated Nrf2 and those with higher levels generally demonstrate poorer prognosis. As such, higher levels of Nrf2 may also serve to activate cancer development, particularly because of the effects on metabolism (54). Nrf2 is therefore likely tightly regulated in cancer.

Activation of Nrf2 has been shown to induce antioxidant response element (ARE)-dependent expression of detoxifying and antioxidant defense proteins, leading to prevention of genome instability. This is believed to inhibit the initiation stage of cancer development, making it a good target for chemoprevention (55). Along with this, it has been seen that Nrf2 is over-expressed in cancer cells, which can lead to cancer progression and resistance to chemotherapeutics. Therefore it is also believed to be a target in cancer treatment (55). It is possible that overexpression of Nrf2 in cancer cells occurs as an epigenetic effect and therefore natural epigenetic modifiers such as polyphenols have been explored for either their adjunctive or their standalone therapy potential. Likewise, some small molecule components of cinnamon have been explored as direct modulators of Nrf2. Both cinnamic aldehyde and methyl-1-cinnamoyl-5-oxo-2-pyrrolidine-carboxylate have been identified as potent Nrf2 activators, cinnamic aldehyde being a natural dietary product, and methyl-1-cinnamoyl-5-oxo-2-pyrrolidine-carboxylate being synthesized (56). High dose cinnamic aldehyde when taken orally was shown to statistically decrease growth of melanoma as well as decrease invasion of the basement membrane in a study performed on mice (57). Along with this, studies have shown that cinnamic aldehyde can be used to prevent melanoma through activation of Nrf2 (56). Nrf2 is a topic that has been heavily researched in cancer due to its antioxidant effects, along with its increased prevalence in some tumors. Many studies have shown cinnamic aldehyde, along with some synthetically created cinnamon-based compounds, greatly increase the activation of Nrf2. Based on the current literature, it is believed that high levels of oral cinnamic aldehyde increases Nrf2. Increased Nrf2 may decrease development of cancer, but increase growth and malignancy of a previously established tumor. However these results should be taken with caution because a study published in Nature found that activated Nrf2 in melanoma cells increased production of cyclooxygenase 2 (COX2) and prostaglandin E2 (PGE2) leading to an “immune-cold tumor environment” which lacks response to many immunotherapies and could increase tumor malignancy (58). It appears that Nrf2 modulation may provide preventative effects, but caution should be used when using as a subjunctive therapy due to the development of favorable tumor microenvironments.

### 3.4 VEGF/HIF1a is decreased by cinnamon treatments

Hypoxia-inducible transcription factor 1 (HIF1) and vascular endothelial growth factor (VEGF) have both been implicated in angiogenesis normally, and within the tumor microenvironment. When a hypoxic environment is created HIF1α is released by the cells, which then upregulates VEGF and angiogenesis occurs. Cancer cells are fast growing, and require new blood vessel growth, therefore HIF1α and VEGF are overexpressed within the tumor microenvironment (59). Cinnamaldehyde, a compound found in cinnamon extract, has been shown to decrease angiogenesis in a dose dependent way. Different cinnamon extracts have been shown to inhibit the HIF1α/VEGF pathway through various mechanisms. Cinnamaldehyde has been shown to decrease HIF1ɑ gene expression, decreasing VEGF, and therefore decreasing angiogenesis and tumor growth. Along with this, procyanidins, an extract of cinnamon, inhibit the action of VEGF on endothelial cells (60). Cinnamon has been studied in both treatment and prevention of cancer. Many anticancer therapies have been targeted at inhibiting VEGF, but they carry a large side effect burden. Cinnamaldehyde shows promise by targeting the HIF1α/VEGF pathway to inhibit tumor growth, with a reduced side effect profile.

Poor prognosis is typically expected in tumors with increased hypoxia. Tumors typically suffer hypoxic conditions that normal cells do not experience, thus hypoxic factors such as HIF1 provide a potential treatment avenue that may result in fewer side effects to healthy cells. Cinnamaldehyde was examined for its effects on the HIF1 pathway and was found to inhibit angiogenesis and metastasis (61). The study also found a decrease in VEGF secretion and VEGF receptor phosphorylation. Protein levels of HIF1 were decreased by cinnamaldehyde via a reduction on protein synthesis (61). HIF1 transcription and translation is tightly regulated in part by the phosphoinositide 3/protein kinase B (Akt)/mechanistic target of rapamycin (mTOR) (PI3K/Akt/mTOR) pathway, which the authors also identified as the likely target of the cinnamaldehyde effect on HIF1 (61). These data suggest that cinnamaldehyde may function in a variety of ways to result in angiogenesis inhibition.

### 3.5 Serine/threonine kinase pathways may be inhibited by cinnamon

Serine/threonine kinases are a group of kinases that regulate a variety of cellular processes including metabolism, signaling, and cell cycle progression. Mitogen-activated protein kinases (MAPK) are one example of a serine/threonine kinases and work by relaying extracellular signals intracellularly and they regulate proliferation, differentiation, motility, and survival (62). The MAPK signaling cascade begins by having a growth factor bind to the growth factor receptor. Activation of these kinases activates rat sarcoma (RAS) and rapidly accelerated fibrosarcoma (RAF) which are small intracellular GTPases, and mutations in this pathway have a high implication of development and growth of malignant tumors (63). Pro-viral insertion in murine lymphomas 1(Pim1) kinase, is a serine/threonine kinase that has been found to be overexpressed in many types of cancer leading to development and progression of the tumor. Pim1 kinase works by phosphorylating the Bad protein leading to inhibition of cellular apoptosis. 2′-Hydroxycinnamicaldehyde (2’HCA), an active compound derived from cinnamon, has been shown to be a direct inhibitor of Pim1 kinase through direct binding of the Pim1 kinase ATP-binding pocket. A study that attempted to treat leukemia and skin cancer lines using 2’HCA tested the concentration of phosphorylated Bad to analyze how 2’HCA inhibition of Pim1 kinase can lead to cancer cell apoptosis. The results showed that there was a direct correlation to concentration of 2’HCA, inhibition of Pim1 kinase, and tumor cell apoptosis (64). This demonstrated that 2’HCA lead to tumor cell apoptosis through inhibition of a kinase. Another study suggested that cinnamon extract inhibited tissue plasminogen activator (TPA) induced phosphorylation of MAPK in a dose dependent manner, leading to an inhibition of angiogenic activity (65). The current research shows that specific serine/threonine kinases can be targets for different cinnamon extracts. By targeting these specific kinases, which have been shown to be mutated in many tumor cell lines, these cinnamon extracts can be anti-angiogenic and increase tumor cell death.

## 4 Cancer prevention

With the advent of increasing healthcare costs, a shift toward emphasis on disease prevention is essential and inevitable. Numerous studies are devoted to determining methods by which to prevent cancer. Given the growing public interest in natural products, a detailed review of the potential preventative effects of cinnamon against cancer is warranted.

### 4.1 Cinnamon positively affects the microbiome

It is well documented that an individual’s diet can alter the person’s microbiome (66, 67). Polyphenols found in cinnamon can be bioactivated in the colon in a microbiome enzyme dependent fashion, highlighting the importance of the maintenance of the gut microbiome (68). Cinnamon essential oil was shown to protect against inflammatory bowel disease in a mouse model of colitis, a condition which greatly increases colon cancer risk (69). Similarly, 100–200 mg/kg bodyweight/day for 2 weeks of cinnamaldehyde was shown to promote intestinal barrier functions in rats as well as decreasing expression of interleukin 6 (IL6) and tumor necrosis factor alpha (TNFα). TNFα typically causes inflammation that can result in pathological changes in the colon (70). This study also demonstrated a recovery of the gut microbiome in the cinnamaldehyde treated rats (70). It is well known that a high fat diet decreases the health of the gut microbiome and increases colon cancer risk; maintaining the gut microbiome is important for the health of the colon (71–74). In normal mice the gut microbiota are altered and the expression of immune response genes are increased with cinnamon supplementation (75). Although cinnamon is likely poised to alter the microbiome in a positive manner and reduce the inflammatory response in the colon, one study, the Polyp Prevention Trial, failed to demonstrate a correlation between certain flavonoid intake and colon cancer (76). The complexity of the overlapping targets of polyphenols, such as those found in cinnamon, make *in vitro* and *in vivo* studies difficult to interpret, but do suggest a strong potential for cinnamon to serve to enhance the microbiome and prevent cancer.

### 4.2 Cinnamon reduced inflammation overall

Overlapping partially with microbiome effects, cinnamon has also demonstrated anti-inflammatory effects (8, 12, 27, 77). A randomized, double blind and controlled clinical trial measuring the plasma levels of NFκB, sirtuins (SIRT), IL6, and TNFα in 20 adult patients with type 2 diabetes treated with cinnamon was completed. The study demonstrated a significant reduction in NFκB, but non-significant reductions in the other markers (27). However, another clinical trial studied the effects of cinnamon on inflammatory markers in women with rheumatoid arthritis and were able to identify a significant decrease in TNFα as well as C reactive protein (78). Another study evaluated the potential of compounds found in cinnamon to inhibit cyclooxygenase-2 and found a modest 0.6–8% inhibition (79). As is commonly the case, *in vitro* studies clearly show a potential for inflammatory reduction, but clinical trials sometimes demonstrate the complexity of the whole system which result in only modest changes in inflammatory factors. None the less, it appears that cinnamon does offer potential as an anti-inflammatory agent by acting on a variety of targets that may be additive *in vivo*. In all cases and reduction in inflammation, even if minor, should play a preventative role. Available cinnamon specific studies are summarized in Table 1.

**Table 1:** Summary of cinnamon study results.

Author	Cancer type	Major conclusions	References number
Liu et al.	breast cancer	Components in cinnamon demonstrates 59 possible targets in treatment of breast cancer. *In vitro* experiments support this by inhibiting cell proliferation, cell migration and invasion as well as promoting apoptosis.	(13)
Tsai et al.	lung cancer	Cinnamon components inhibit invasion of A549 cells via a proposed mechanism of decreasing the signaling of MAPK and PI3K/Akt as well as inactivating NFκB and AP1.	(20)
Gopalakrishnan	prostate cancer	Cinnamon components decrease cell proliferation and antiapoptotic and angiogenic markers.	(18)
Davari	N/A	Cinnamon components create a significant decrease in NFκB levels	(27)
Schink et al.	N/A	Cinnamon extract exhibits an anti-inflammatory effect, including altering levels if IL8 and NFκB.	(53)
Naghiaee et al.	N/A	Cinnamaldehyde reduced expression of MiRs 29a, 223, and 320. Expression of miR 26b was up-regulated.	(32)
Kwon et al.	various types	Anti-tumor activity of cinnamon extracts is directly linked to enhanced pro-apoptotic activity and inhibition of NFκB and AP1 activities.	(51, 52)
Wondrak et al.	skin cancer	Cinnamon-based Nrf2 activators decreased oxidative stress and protected against photo-oxidative induction of apoptosis in skin cells.	(56)
Cabello et al.	melanoma	Cinnamic aldehyde impairs proliferation, invasiveness and tumor growth.	(57)
Zhang et al.	ovarian cancer	Cinnamaldehyde inhibits VEGF expression.	(60)
Patra et al.	skin cancer	Cinnamaldehyde decreases angiogenesis and metastasis by inhibiting HIF1α.	(61)
Kim et al.	leukemia and skin cancer	Cinnamaldehyde demonstrates anticancer potential by targeting Pim1 kinase.	(64)
Bansode et al.	N/A	Cinnamon extract demonstrated antiangiogenic activity.	(65)
Li et al.	N/A	Cinnamon essential oil inhibited development of colitis and improved the diversity of intestinal microbiota.	(69)
Qi et al.	N/A	Cinnamaldehyde attenuates the activation of NFκB signaling and suppresses inflammatory cytokines as well as remodeling the gut microbiome.	(70)
Kim et al.	N/A	Cinnamon increased gene expression of genes necessary for defense against gut bacteria and increased gene expression for genes involved in lipid absorption.	(75)
Shishebor et al.	N/A	Cinnamon decreased the serum levels of CRP and TNFα.	(78)
Willis et al.	colon cancer	Cinnamon exhibited anti-inflammatory potential by inhibiting COX2.	(79)

MAPK, mitogen-activated protein kinases; PI3K/Akt/mTOR, phosphoinositide 3/protein kinase B (Akt)/mechanistic target of rapamycin (mTOR); NFκB, Nuclear Factor kappa B; AP1, activator protein 1; IL8, interleukin 8; MiR, microRNA; Nrf2, Nuclear factor erythroid related factor 2; VEGF, vascular endothelial growth factor; HIF1α, Hypoxia-inducible transcription factor 1 alpha; Pim1, Pro-viral insertion in murine lymphomas 1; CRP, C reactive protein; TNFα, tumor necrosis factor alpha; COX2, cyclooxygenase 2.

## 5 Bioavailability

As with any drug or herbal supplement, absorption, and distribution are important factors to consider when using cinnamon as an anticancer therapy. Cinnamon therapies must be able to reach the appropriate site of action after administration in order to be effective agents. Cinnamaldehyde, one of the main compounds in cinnamon with anticancer activity has demonstrated poor water solubility which could impair its oral absorption and distribution in blood. Therefore, some researchers have investigated drug delivery systems that could enhance the oral bioavailability of cinnamon.

Superparamagnetic iron oxide nanoparticles (SPION) is a novel drug delivery method currently being studied for various chemotherapies and could be applied to cinnamon. SPION consists of a core of iron oxide nanoparticles that are covered by a coating that can be made of various organic compounds. The coating allows for water solubility and to assist with attaching the iron oxide to therapeutic agents such as antibodies and drugs. SPIONs can be influenced by external magnets outside of the patient’s body so that they accumulate near a target tissue and enhance the bioavailability of the therapeutic agent. This is beneficial for getting a drug to reach a target that it would otherwise have difficulty finding and it can reduce the amount of drug that accumulates in unwanted areas which mitigates side effects (80).

Another possible benefit of using SPION drug delivery systems for cinnamon treatments is through the use of magnetic hyperthermia. An alternating magnetic field can be used to generate heat within the nanoparticles which can induce cell death. This method has the benefit of being selective because cancer cells are more sensitive to death by hyperthermia than healthy cells which means there is minimal collateral damage from this technique (80). A cinnamon treatment tagged with a SPION could therefore be a double threat to cancer cells as the enhanced, selective drug delivery and magnetic hyperthermia capabilities can work together to kill tumors through chemical and physical means.

## 6 Conclusion

Many of the proposed mechanisms of cinnamon interaction overlap with each other. For example, numerous studies show a link between cancer and NFκB or related regulators such as IκB proteins and VEGF and TNFα. Furthermore, studies summarized demonstrate an effect of cinnamon on these specific regulators. One clear conclusion is that cinnamon reduced inflammation and reduced inflammation, through a variety of targets, reduced cancer. Although this is a loose connection, it is a significant pathway overlap that should be further studied. Limitations in the studies exist, reviews could cover even more papers, but overall there is a clear conclusion that the potential of cinnamon to effect cancer pathways exists and must be further studied. The intent of this review is to stimulate a desire to develop research questions in this field and establish well-designed studies to supplement the available literature.

Several of the articles cited cover studies with cinnamon, but due to the limited number of cinnamon and cancer studies specifically, some studies linking the pathways to cancer are included. Many studies elucidate the mechanism of inflammation affected by micro RNAs for example, while other studies link cinnamon components to the micro RNA production. These links were made with the intention of demonstrating potential areas of cinnamon influence in cancer and are in no way intended to over interpret. This review was conducted to demonstrate potential areas for cinnamon studies in cancer that should clearly initiate under *in vitro* conditions. As more specific studies are conducted to elucidate cinnamon specific pathways, *in vivo* studies could be proposed. As summarized in this review, many studies are *in vitro* but do demonstrate a potential for theoretical anticancer activity. Additional animal studies and eventually clinical studies are needed to demonstrate if the potential seen with these compounds does in fact result in improved prognosis for patients or decreased cancer development. As with all chronic diseases, including cancer, clinical studies must be carefully designed to make sure placebo groups are not disadvantaged if clinical results demonstrate the effectiveness of cinnamon in cancer treatment and prevention. Additional safety studies are also necessary in order to ensure that treatment levels of active compounds are within an efficacious yet non-toxic range. In recent years, more animal studies have been undertaken, but these need to be expanded and developed into potential clinical studies for completeness.

The field of miRNA has exploded in the last decade and miRNAs related to a variety of disease states has shed light on the previously unexplained causes of disease. Cinnamon has been shown to affect a number of miRNA molecules in insulin resistance and in obesity (32, 33). Examining the effects of cinnamon components on miRNA involved in cancer is one necessary area of research that needs expanded to complement the CAM field.

As with all plant-based treatments, it is important to consider potential risks and toxicity. A study published in the Journal of the American Heart Association explored the correlations between specific spices, including cinnamon, and mortality. The study demonstrated a reduced mortality in patients taking turmeric or saffron but did not demonstrate any changes in mortality related to cinnamon (81). Although this study did not find any adverse effects it does highlight the importance of conducting such evaluations. Cinnamon does contain high levels of coumarin that have the potential to result in liver damage (82–84). Of importance is that high coumarin levels may induce cancer and has been observed in animal studies (85). Although at high levels this development of cancer has been observed in animals, additional studies have not been completed. Furthermore, numerous recent studies suggest coumarin at the levels tested possess anti-cancer activity (86–89).
